# Estimated Annual Number of HIV Infections ─ United States, 1981–2019

**DOI:** 10.15585/mmwr.mm7022a1

**Published:** 2021-06-04

**Authors:** Karin A. Bosh, H. Irene Hall, Laura Eastham, Demetre C. Daskalakis, Jonathan H. Mermin

**Affiliations:** ^1^Division of HIV/AIDS Prevention, National Center for HIV/AIDS, Viral Hepatitis, STD, and TB Prevention, CDC; ^2^National Center for HIV/AIDS, Viral Hepatitis, STD, and TB Prevention, CDC.

The first cases of *Pneumocystis carinii* (*jirovecii*) pneumonia among young men, which were subsequently linked to HIV infection, were reported in the *MMWR* on June 5, 1981 (*1*). At year-end 2019, an estimated 1.2 million persons in the United States were living with HIV infection (*2*). Using data reported to the National HIV Surveillance System, CDC estimated the annual number of new HIV infections (incidence) among persons aged ≥13 years in the United States during 1981–2019. Estimated annual HIV incidence increased from 20,000 infections in 1981 to a peak of 130,400 infections in 1984 and 1985. Incidence was relatively stable during 1991–2007, with approximately 50,000–58,000 infections annually, and then decreased in recent years to 34,800 infections in 2019. The majority of infections continue to be attributable to male-to-male sexual contact (63% in 1981 and 66% in 2019). Over time, the proportion of HIV infections has increased among Black/African American (Black) persons (from 29% in 1981 to 41% in 2019) and among Hispanic/Latino persons (from 16% in 1981 to 29% in 2019). Despite the lack of a cure or a vaccine, today’s HIV prevention tools, including HIV testing, prompt and sustained treatment, preexposure prophylaxis, and comprehensive syringe service programs, provide an opportunity to substantially decrease new HIV infections. Intensifying efforts to implement these strategies equitably could accelerate declines in HIV transmission, morbidity, and mortality and reduce disparities.

To estimate annual HIV incidence among persons aged ≥13 years in the United States during 1981–2019, CDC applied mathematical modeling to data reported to the National HIV Surveillance System. Three eras of HIV incidence estimates were used based on changes in methodology and available data (*3*,*4*).* The cumulative number of HIV infections over the period was estimated by summing annual incidence estimates. The distributions of HIV incidence were compared overall and by sex at birth, race/ethnicity, and transmission category for the period examined at the beginning (1981), at the peak number of annual infections (1984–1985), and at the end of the study period (2019). Trends in the annual number of HIV infections over the entire period were assessed for selected racial/ethnic groups and transmission categories.^†^ For racial/ethnic groups, only trends among Black, Hispanic/Latino, and White persons were described.^§^ Increases or decreases in the numbers and proportions are reported for relative changes of ≥5%.

During 1981–2019, there were an estimated 2.2 million new HIV infections among persons aged ≥13 years in the United States. The estimated number of infections increased from 20,000 in 1981 (Figure 1) to 130,400 in 1984 and 1985, then declined rapidly to between 84,200 and 84,800 annually during 1986–1990. HIV incidence remained relatively stable from 1991 to 2007, with about 50,000 to 58,000 infections per year, and declined in recent years to an estimated 34,800 in 2019. HIV incidence decreased by 73% from the highest annual number of infections (130,400 in 1984 and 1985) to 34,800 in 2019.

**FIGURE 1:**
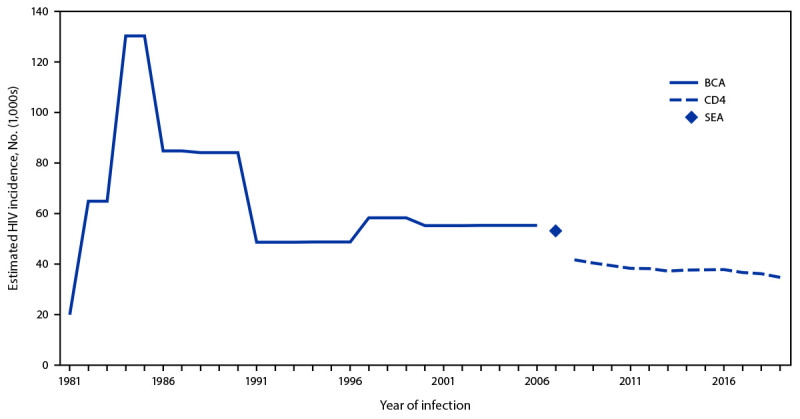
Estimated HIV incidence* among persons aged ≥13 years — United States, 1981–2019 **Abbreviations:** BCA = back-calculation approach; CD4 = CD4+ T-lymphocyte model; SEA = stratified extrapolation approach. * HIV incidence estimates for 1981–2006 were derived from the extended BCA applied to HIV surveillance data reported to CDC through June 2007. HIV incidence in 2007 was estimated using the SEA applied to HIV surveillance data reported to CDC through June 2011. HIV incidence estimates during 2008–2019 were derived from the CD4 model applied to HIV surveillance data reported to CDC through December 2020.

A larger proportion of infections occurred among females in 2019 (18%) than did in 1981 (8%) or in 1984–1985 (12%). The number of HIV infections among White persons decreased during 1985–2019 (Table) (Figure 2) and the proportion of infections among White persons decreased from 56% in 1981 to 25% in 2019. The number of infections among Black persons increased during 1981–1990 and then decreased through 2019. In 1988, the number of infections among Black persons surpassed the number among White persons and remained higher than in any other racial/ethnic group through 2019. Black persons accounted for 29% of infections in 1981, 30% of infections in 1984–1985, and 41% of infections in 2019. Hispanic/Latino persons represented 16% of infections in 1981, 14% of infections in 1984–1985, and 29% of infections in 2019.

**TABLE:** Estimated HIV incidence among persons aged ≥13 years, by selected characteristics — United States, 1981, 1984–1985, and 2019

Characteristic	No. (%)		
	1981*	1984–1985*	2019^†^
**Sex at birth**			
Male	18,600 (93)	115,500 (89)	28,400 (82)
Female	1,500 (8)	15,100 (12)	6,400 (18)
**Race/Ethnicity**			
American Indian/Alaska Native	0 (—)	400 (0)	230^§^ (1^§^)
Asian^¶^	N/A	N/A	550 (2)
Asian/Pacific Islander^¶^	0 (—)	900 (1)	N/A
Black/African American	5,800 (29)	38,800 (30)	14,300 (41)
Hispanic/Latino**	3,100 (16)	18,200 (14)	10,200 (29)
Native Hawaiian/Other Pacific Islander^¶^	N/A	N/A	─^††^
White	11,100 (56)	72,100 (55)	8,600 (25)
Multiple races^¶^	N/A	N/A	900 (3)
**Transmission category^§§^**			
Male-to-male sexual contact	12,500 (63)	75,800 (58)	23,100 (66)
Injection drug use	4,400 (22)	32,000 (25)	2,500 (7)
Male-to-male sexual contact and injection drug use	2,400 (12)	11,400 (9)	1,400 (4)
Heterosexual contact^¶¶^	400 (2)	8,000 (6)	7,800 (22)
**Total**	**20,000 (100)**	**130,400 (100)**	**34,800 (100)**

**Abbreviation:** N/A = not applicable.

* Estimates derived from the extended back-calculation model applied to HIV surveillance data reported to CDC through June 2007. Estimates are rounded to the nearest 100 and subtotals for sex at birth and transmission category do not sum to the overall total. Percentages were calculated using rounded estimates and might not sum to 100%. The highest numbers of annual HIV infections during 1981–2019 occurred in 1984−1985.

^†^ Estimates derived from the CD4+ T-lymphocyte model applied to HIV surveillance and CD4 data reported to CDC through December 2020. Estimates rounded to the nearest 100 for estimates >1,000 and to the nearest 10 for estimates ≤1,000 to reflect model uncertainty. Percentages were calculated using rounded estimates and might not sum to 100%.

^§^ Estimate with a relative standard error of 30%–50%; estimate should be used with caution.

^¶^ HIV surveillance data collection requirements for race and ethnicity changed in 2003 to align with the revisions to the standards for the classification of federal data on race and ethnicity mandated by the Office of Management and Budget. In 1981, multiple racial categories could not be collected for a person and data for all Asian and Pacific Islander persons were collected as a single racial category. In 2019, multiple racial categories could be collected for a person and data for Asian persons were collected separately from Native Hawaiian and other Pacific Islander persons.

** Hispanic/Latino persons can be of any race.

^††^ Estimates with a relative standard error of >50% are not shown.

^§§^ Transmission categories assigned on the basis of sex at birth and include transgender persons. Data by transmission category have been adjusted to account for missing risk-factor information.

^¶¶^ Heterosexual contact with a person known to have, or with a risk factor for, HIV infection.

**FIGURE 2:**
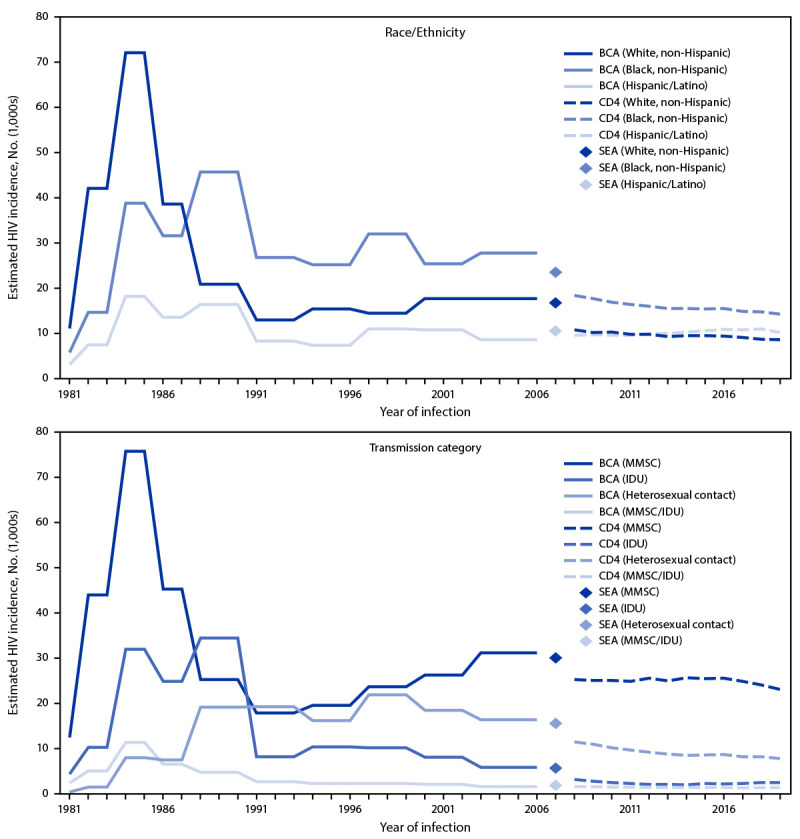
Estimated HIV incidence* among persons aged ≥13 years, by selected race/ethnicity^†^ and transmission category^§^ — United States, 1981–2019 **Abbreviations:** BCA = back-calculation approach; CD4 = CD4+ T-lymphocyte model; IDU = injection drug use; MMSC = male-to-male sexual contact; SEA = stratified extrapolation approach. * HIV incidence estimates for 1981–2006 were derived from the extended BCA applied to HIV surveillance data reported to CDC through June 2007. HIV incidence in 2007 was estimated using the SEA applied to HIV surveillance data reported to CDC through June 2011. HIV incidence estimates during 2008–2019 were derived from the CD4 model applied to HIV surveillance data reported to CDC through December 2020. ^†^ Hispanic/Latino persons can be of any race. ^§^ Transmission categories assigned on the basis of sex at birth and include transgender persons. Data by transmission category have been adjusted to account for missing risk-factor information.

Male-to-male sexual contact accounted for more than one half of infections in all years except during 1988–2002, when infections attributed to heterosexual contact increased. The proportion of infections attributed to male-to-male sexual contact or male-to-male sexual contact and injection drug use was 75% in 1981, 67% in 1984–1985, and 70% in 2019. The proportion of infections attributed to heterosexual contact was higher in 2019 (22%) than in 1981 (2%) or in 1984–1985 (6%), whereas the proportion of infections among persons who inject drugs was lower in 2019 (7%) than in 1981 (22%) or in 1984–1985 (25%).

## Discussion

Since the peak of the HIV epidemic, models show that incidence decreased substantially, from 130,400 in the mid-1980s to 34,800 in 2019. However, disparities continue, and some have worsened over time. For example, in 2019, Black persons accounted for 41% of new HIV infections but for only 12% of the U.S. population.^¶^ Hispanic/Latino persons accounted for 29% of new HIV infections in 2019, although they represent 17% of the population. Infections among men who have sex with men, including those who inject drugs, accounted for 70% of infections in 2019, but men who have sex with men account for only an estimated 2% of the population (*5*). Transgender women also are significantly at risk for HIV infection; a recent CDC report found that four in 10 transgender women surveyed in seven major U.S. cities have HIV infection.**

During the past 4 decades, the largest relative reduction in HIV incidence occurred among persons who inject drugs; incidence decreased 93% from the highest annual number, 34,500 in 1988–1990, to 2,500 in 2019. Incidence has not decreased during the past decade, likely, in part, because of the opioid epidemic, which is associated with increased drug use and needle sharing.^††^^.^ The decrease in injection drug use–associated HIV infections followed the implementation of syringe service programs, which have been widely shown to be effective in preventing transmission of HIV.^§§^However, syringe service programs are not available in all areas.

A major factor in the reduction of HIV infection has been the participation of persons that have or are at risk for HIV, community activists, scientists, politicians, and public health officials in steering the national and community response to this epidemic (*6*). Communication and collaboration between these groups has resulted in a more robust, equitable, and effective response.

Reductions in incidence during 1981–2019 likely reflect increased availability of and access to HIV diagnostics, including high throughput laboratory-based technology, point-of-care tests, and over-the-counter test kits; implementation of routine HIV screening and antiretroviral therapy regardless of immune status or disease stage; and programmatic efforts to increase linkage to care, re-engagement in care, behavior change, use of pre- and postexposure prophylaxis, and syringe service programs. Efforts to increase availability of and access to HIV diagnostics have led to an increase in the proportion of estimated persons living with HIV who know their status.^¶¶^ The effectiveness of antiretroviral therapy has improved substantially, and sustained viral suppression prevents sexual transmission of HIV. Today, persons who receive an HIV diagnosis soon after infection and who maintain viral suppression have a nearly normal lifespan (*7*).

Preexposure prophylaxis (i.e., antiretroviral medication taken before potential exposure to prevent infection) has considerable promise in further decreasing HIV incidence, and medication is >99% effective in preventing acquisition of HIV when taken as prescribed. However, only 23% of persons who could benefit from preexposure prophylaxis were using it in 2019 (*2*). Racial and ethnic disparities in preexposure prophylaxis prescribing are pronounced; preexposure prophylaxis was prescribed for 63% of the estimated number of White persons who could benefit from it but was prescribed for only 8% of Black persons and 14% of Hispanic/Latino persons who could benefit from it. Prevention tools are increasingly effective, but they need to reach the populations most affected.

Gaps in service access and other social and economic determinants, including stigma and discrimination, are ongoing obstacles that hinder adherence to antiretroviral therapy and viral suppression, and thereby perpetuate disparities. The Ryan White Care Program, which provides comprehensive HIV primary medical care, support services, and medications for low-income persons with HIV infection, is an example of how an integrated program can reduce disparities in viral suppression across populations (*8*).

Underlying causes for many disparities highlight the importance of social and economic determinants of health. Efforts to end the HIV epidemic that center on accelerating implementation of treatment and prevention technology can do so more effectively by focusing on root social causes of these well-documented HIV-related disparities. These systemic barriers, which include systemic racism, poverty, homelessness, discrimination, homophobia, and transphobia, impede access to testing, treatment, and prevention services and drive inequity (*9*).

The findings in this report are subject to at least three limitations. First, three mathematical models were used to estimate incidence over different portions of the analysis period. Each model is subject to assumptions that might result in different estimates of incidence; however, a previous analysis of HIV incidence from 2008–2013 using three models found incidence trends were generally corroborated across the models (*10*). Second, the back-calculation approach, which was used to estimate incidence for 1981–2006, did not produce single-year estimates, but rather average estimates over a 2- to 4-year interval. Therefore, year-to-year changes in HIV incidence cannot be assessed through 2006. Finally, estimates derived from all three models are subject to uncertainty attributable to assumptions such as accurate diagnosis dates, accuracy of models to identify diagnosis delays, and the impact of migration. However, trend data comparing subpopulations is likely to be robust for each period examined.

The prevention tools available today, including HIV testing, prompt and sustained treatment, preexposure prophylaxis, and comprehensive syringe service programs, as well as new technologies being developed, such as long-acting antiretroviral agents, self-testing, and telemedicine, provide an opportunity to substantially decrease new HIV infections.*** Ongoing priorities should include maximizing critical partnerships, implementing treatment and prevention services at scale, and ensuring a focus on decreasing disparities. Ending the HIV epidemic requires addressing health disparities. Equitable implementation of prevention tools to diagnose HIV infection early, treat persons with HIV to rapidly achieve viral suppression, and link persons to preventive services to reduce new transmissions will hasten the decrease in HIV incidence.

**Summary**

**What is already known about this topic?**

HIV incidence decreased from the 1980s through 2019.

**What is added by this report?**

HIV incidence decreased by 73% from the highest number of infections (130,400) in 1984 and 1985 to 34,800 in 2019. A larger proportion of infections was among Black/African American and Hispanic/Latino persons in 2019 than in 1981.

**What are the implications for public health practice?**

HIV treatment and prevention services should be tailored to the most affected communities and their service providers and address social and economic obstacles contributing to HIV-related health disparities. Ending the HIV epidemic requires equitable implementation of prevention tools to diagnose HIV infection early, treat persons with HIV to rapidly achieve viral suppression, and link persons to preventive services.

## Footnotes

* HIV incidence estimates for 1981–2006 were derived from the extended back-calculation approach applied to HIV surveillance data reported to CDC through June 2007. HIV incidence in 2007 was estimated using the stratified extrapolation approach applied to HIV surveillance data reported to CDC through June 2011 (https://www.cdc.gov/hiv/pdf/library/reports/surveillance/cdc-hiv-surveillance-supplemental-report-vol-17-4.pdf). HIV incidence estimates during 2008–2019 were derived from the CD4 model applied to HIV surveillance data reported to CDC through December 2020.

† Transmission categories were assigned on the basis of sex at birth, regardless of gender identity.

§ Trends were not assessed for racial/ethnic groups other than White, Black, and Hispanic/Latino because of changes in data collection that were required in 2003 to align with revised standards for classification of federal data on race and ethnicity for other racial categories, as well as the small number of infections.

¶
https://www.census.gov/programs-surveys/popest/data/data-sets.html

**
https://www.cdc.gov/hiv/pdf/library/reports/surveillance/cdc-hiv-surveillance-special-report-number-27.pdf

††
https://www.cdc.gov/pwid/opioid-use.html

§§
https://www.federalregister.gov/documents/2011/02/23/2011-3990/determination-that-a-demonstration-needle-exchange-program-would-be-effective-in-reducing-drug-abuse

¶¶
https://www.cdc.gov/nchhstp/atlas/index.htm

***
https://www.cdc.gov/HIV/basics/prevention.html
